# MXene/Cuttlefish-Ink Nanoparticles Incorporated Dual-Purification Sponge for Solar-Driven Oily Wastewater and Microplastic Remediation

**DOI:** 10.3390/polym18030324

**Published:** 2026-01-26

**Authors:** Huixuan Sun, Qirui Gong, Lihong Fan, Shilin Tian, Shiyuan Yao, Guangxu Wang, Sasha You, Wei Zhang

**Affiliations:** 1 Sanya Science and Education Innovation Park of Wuhan University of Technology, Sanya 572024, China; 290406@whut.edu.cn (H.S.); 348337@whut.edu.cn (S.Y.); 2 School of Resources and Environmental Engineering, Wuhan University of Technology, Wuhan 430070, China; 3 School of Chemistry, Chemical Engineering and Life Sciences, Wuhan University of Technology, Wuhan 430070, China; 335117@whut.edu.cn (Q.G.); tsl@whut.edu.cn (S.T.); 348334@whut.edu.cn (G.W.); 358846@whut.cn (S.Y.); 346372@whut.edu.cn (W.Z.)

**Keywords:** flexible polyurethane sponge, superhydrophobic/super oleophilic, oil/water separation, crude oil absorption, microplastics adsorption

## Abstract

The escalating severity of microplastic pollution and oily wastewater discharge has intensified the demand for recyclable, multifunctional, and environmentally benign materials. In this study, we present a composite polyurethane (PU) sponge constructed through the synergistic integration of cuttlefish-ink nanoparticles (CINPs), Ti_3_C_2_T_X_ MXene, and polydimethylsiloxane (PDMS). The synergistic CINP@MXene framework imparts high photothermal conversion efficiency and structural stability, while the PDMS coating confers superhydrophobicity. The resulting sponge demonstrates efficient oil absorption and oil–water separation capabilities, alongside a stable photothermal response, achieving a temperature of 84.1 °C within 10 s under 1.5 Sun irradiation. Notably, the sponge absorbed approximately 0.05 g of crude oil within 10 s, the saturated absorption capacity of crude oil under 1.5 solar days was 24.52 g/g, and the adsorption rate of 5 g crude oil within 4 min was 91.4%. Furthermore, it exhibits remarkable adsorption performance toward common microplastics and nanoplastics. Overall, the CINPs@MXene/PU/PDMS sponge represents a versatile and scalable platform with significant potential for addressing challenges in oily wastewater treatment, solar-assisted oil recovery, and microplastic remediation, thereby contributing to sustainable environmental protection efforts.

## 1. Introduction

The escalating challenge of microplastic pollution represents a major global threat to aquatic environments [1,2]. Owing to their high stability and mobility, microplastics accumulate persistently in oceans, lakes, and other water bodies, endangering aquatic ecological security and organismal health [3,4,5,6]. Concurrently, rapid urbanization and industrialization have exacerbated water pollution, particularly through the frequent and complex discharge of oily wastewater [7,8]. Such pollution, deriving mainly from industrial effluents, domestic sewage, and crude oil spills, inflicts long-term harm on ecosystems and poses direct risks to human health. It is noteworthy that most types of microplastics and oil pollutants share strongly hydrophobic characteristics [9,10,11]. Consequently, the development of materials with superior hydrophobic properties has become a common strategic approach for achieving efficient removal of both pollutant categories.

While sorption is a simple and efficient remediation strategy, current materials face significant limitations [12,13]. Traditional methods like in situ combustion or filtration often suffer from low efficiency, secondary pollution, and high energy consumption [14]. Although advanced sorbents have been developed, they typically exhibit single functionalities and critical drawbacks. For instance, graphene or cellulose-based aerogels, despite high oil capacity, are structurally fragile for repeated use in harsh environments [15,16]. Similarly, polyurethane (PU) sponges, while being a promising scaffold due to their porous structure and low cost, inherently suffer from poor oil/water selectivity and, critically, high flammability [17]. Furthermore, existing modification efforts often introduce trade-offs. Therefore, there is an urgent need to develop a single, robust material that synergistically integrates efficient oil/water separation, effective microplastic adsorption, and intrinsic flame retardancy to overcome these multifaceted challenges [18].

**Figure 1:**
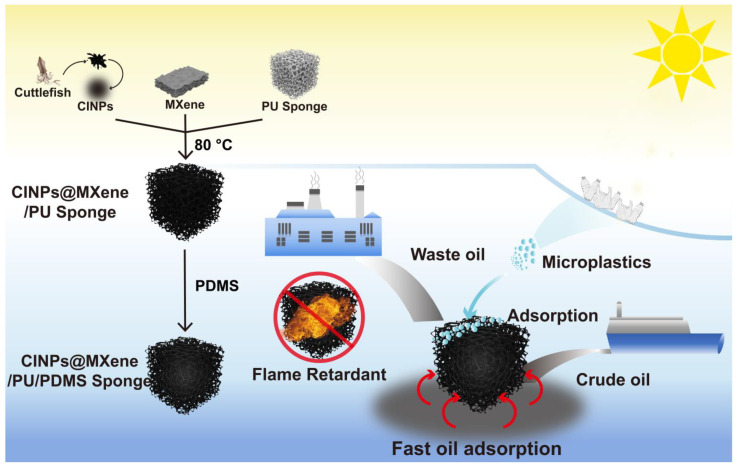
Schematic illustration of the synthesis and multifunctional performance of the CINPs@MXene/PU/PDMS sponge, demonstrating its capabilities in efficient photothermal enhanced crude oil recovery, adsorption of microplastics, and flame retardancy.

In this study, we report a multifunctional intelligent composite polyurethane (PU) sponge designed by combining naturally derived cuttlefish ink nanoparticles (CINPs), artificially synthesized two-dimensional transition metal carbide Ti_3_C_2_T_X_ MXene, and the low-surface-energy polymer polydimethylsiloxane (PDMS) (Figure 1). CINPs, with a photothermal conversion efficiency of up to 40% [19,20], outperform conventional melanin-like nanoparticles (~29%), driving efficient oil absorption. The antioxidant properties of CINPs delay MXene oxidation, enhancing stability. MXene boosts mechanical strength and synergistically improves photothermal responsiveness and durability. Its flame-retardant properties also enhance the sponge’s thermal stability and safety. A hierarchical micro/nanostructured surface, formed by depositing CINPs@MXene onto the PU scaffold, increases hydrophobicity. PDMS coating further confers superhydrophobicity, mechanical robustness, and interfacial stability, while enabling effective adsorption of microplastic particles. This innovative composite sponge, combining superhydrophobicity, superoleophilicity, rapid photothermal responsiveness, efficient crude oil absorption and microplastic adsorption, and flame retardancy, shows great potential in treating complex oil pollution and microplastic contamination. The study presents a nature-inspired hybrid design strategy for developing high-performance, safe, and sustainable materials for oil/water separation and microplastic remediation, offering robust support for oily wastewater treatment, marine emergency responses, and microplastic pollution control.

## 2. Materials and Methods

### 2.1. Materials

PU sponge was collected from Shifeng New Materials Co., Ltd., Xianning, Hubei, China. A natural cuttlefish sac was purchased from Moyuanwai Food Technology Co., Ltd., Sanmenxia, Henan, China. Anhydrous ethanol (EtOH, 95%), N, n-dimethylformamide (DMF, AR, ≥99.5%), n-hexane (AR, ≥97%), ethyl acetate (AR, ≥99.5%), silicone oil, petroleum ether, xylene (AR, ≥99%), chloroform (AR, ≥99%), carbon tetrachloride (AR, ≥99.5%), and dichloromethane (AR, ≥99.5%) were purchased from Sinopharm Chemical Reagent Co., Ltd., Shanghai, China. Ti_3_AlC_2_ MAX was purchased from Foshan Xinxi Technology Co., Ltd., Foshan, Guangdong, China. Lithium fluoride (LiF, AR, 99%) and dimethyl sulfoxide (DMSO, AR, 99%) were purchased from Macklin Biochemical Co., Ltd., Shanghai, China. Polydimethylsiloxane (PDMS) was purchased from Dow Corning Corporation. Peanut oil was purchased from Luhua Group Co., Ltd., Yantai, Shandong, China. The vacuum pump oil was purchased from Jingzuan Lubricating Oil Co., Ltd., Dongguan, Guangdong, China. Microplastics (Polyethene terephthalate, polystyrene, polyethene, polyvinyl chloride, and polymethyl methacrylate) and nanoplastics (Polystyrene, 100 nm) were purchased from Tomicro Biotech Co., Ltd., Shanghai, China.

### 2.2. Prepration of CINPs, MXene, CINPs@ MXene, and CINPs@MXene/PU/PDMS Sponge

#### 2.2.1. Preparation of CINPs

The cuttlefish ink was collected from the cuttlefish sac in a centrifuge tube and centrifuged at 10,000 rpm for 10 min to remove the supernatant. Subsequently, the precipitate was washed multiple times by centrifugation with ethanol and deionized water, dried in a vacuum drying oven at 90 °C for 12 h, and then ground in a quartz mortar to obtain CINPs [19].

#### 2.2.2. Preparation of MXene

Ti_3_C_2_T_X_ MXene was carried out via a chemical etching method. First, 10 mL of deionized water was mixed with 10 mL of concentrated HCl and 1 g of LiF, and the mixture was stirred for 15 min. Next, 1 g of the ceramic Ti_3_AlC_2_ MAX was gradually added to the mixed solution in several portions. The resulting solution was then stirred in an oil bath at 55 °C for 24 h. After the reaction, the solution was repeatedly centrifuged and washed with deionized water, combined with ultrasonic treatment, until the pH reached 7. The centrifuged product was dried in a vacuum oven at 40 °C for 12 h to obtain multilayer Ti_3_C_2_T_X_ MXene powder. This powder was subsequently mixed with DMSO at a 1:10 weight ratio and sonicated for 2 h. Following centrifugation to remove the supernatant, the remaining product was washed several times with anhydrous ethanol and dried to yield a few-layer Ti_3_C_2_T_X_ MXene powder [21,22].

#### 2.2.3. Preparation of CINPs@MXene

**Figure 2:**
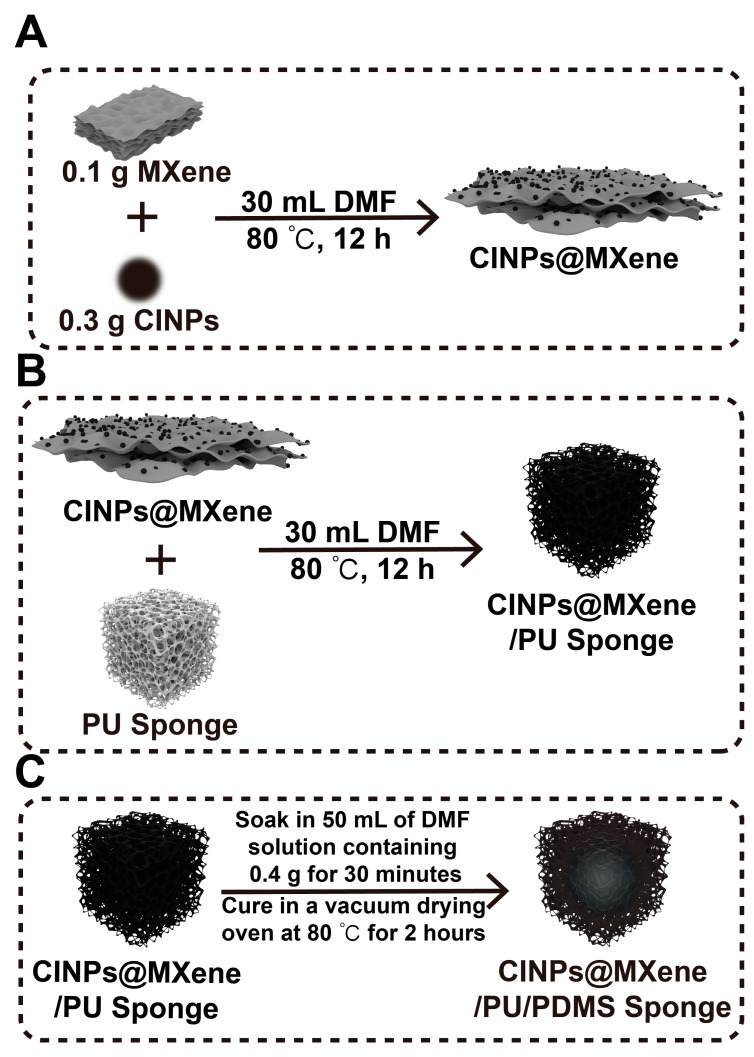
Hematic diagram of the preparation process. (**A**) Synthesis of the CINPs@MXene. (**B**) Fabrication of the CINPs@MXene/PU sponge. (**C**) Preparation of the CINPs@MXene/PU/PDMS sponge via PDMS coating.

To prepare CINPs@MXene, first disperse 0.3 g of CINPs and 0.1 g of MXene in 30 mL of DMF, then ultrasonicate for 30 min for uniformity. Transfer the dispersion to a high-pressure reactor and maintain at 80 °C for 12 h. After the reaction and cooling to room temperature, rinse the precipitate repeatedly with DMF to eliminate residual reactants. Finally, dry the precipitate in a vacuum oven to obtain CINPs@MXene. The schematic illustration for the fabrication of CINPs@MXene was shown in Figure 2A.

#### 2.2.4. Preparation of CINPs@MXene/PU/PDMS Sponge

The PU sponge was ultrasonically cleaned with ethanol and deionized water, then dried at 50 °C. A 2 cm × 2 cm × 2 cm sponge was mixed with CINPs@MXene in 30 mL DMF, ultrasonicated for 30 min, and reacted at 80 °C for 12 h in a 100 mL reactor. After cooling, the sponge was dried at 50 °C in a vacuum oven.

To construct a superhydrophobic surface, 0.4 g of PDMS (containing 10 wt% curing agent) was dispersed in 50 mL of DMF to form a uniform solution. The above-mentioned modified sponge was immersed in the PDMS solution and ultrasonically treated for 30 min to ensure uniform coating by PDMS. Finally, the sponge was placed in a vacuum drying oven and cured at 80 °C for 2 h to obtain the CINPs@MXene/PU/PDMS sponge. The schematic illustration for the fabrication of CINPs@MXene/PU Sponge and CINPs@MXene/PU/PDMS Sponge is shown in Figure 2B,C.

We systematically prepared four CINPs@MXene/PU/PDMS composite sponges with different CINPs@MXene loading ratios, expressed as a weight percentage relative to the DMF solvent. The specific doping concentrations are summarized in Table 1.

### 2.3. Characterization

Fourier transform infrared spectroscopy (FT-IR, Nicolet6700, Thermo Fisher, Waltham, MA, USA) was employed to analyze the chemical composition and functional group evolution of the materials. For the powdered samples (CINPs, MXene, and CINPs@MXene), spectra were acquired using the KBr pellet method. For the solid sponge samples (PU Sponge, CINPs@MXene/PU Sponge, and CINPs@MXene/PU/PDMS Sponge), the attenuated total reflectance (ATR) mode was utilized to directly characterize the materials in their native state. All spectra were recorded in the range of 4000–500 cm^−1^. X-ray Diffraction (XRD, Empyrean, PANalytical B.V., Almelo, Netherlands) was used to analyze the crystal structures of CINPs, MXene, and CINPs@MXene. The surface chemical composition and electronic states were analyzed by X-ray photoelectron spectroscopy (XPS, K-Alpha, Thermo Fisher Scientific, Waltham, MA, USA) using a monochromatic Al Kα radiation source (1486.6 eV). The analysis was performed under a vacuum of approximately 10^−7^ Pa. The survey spectra were acquired with a pass energy of 100 eV, while high-resolution spectra were collected at a pass energy of 30 eV. All binding energies were calibrated using the adventitious C 1s peak at 284.8 eV. The particle size analyzer (ZS90, Malvern, Worcestershire, UK) was used to determine the hydrated particle size of CINPs. The transmission electron microscope (TEM, JEM-1400Plus, JEOL, Tokyo, Japan) was used to examine the micromorphology and particle size of CINPs, MXene, and CINPs@MXene. The scanning electron microscope (SEM, Gemini 300, Zeiss, Oberkochen, Germany) was utilized to reveal the structural morphology of the PU sponge, CINPs@MXene/PU sponge, and CINPs@MXene/PU/PDMS sponge. Prior to imaging, the non-conductive sponge samples were mounted on conductive adhesive tape and sputter-coated with a thin gold layer for 60 s at a current of 10 mA using an ion sputter coater (MC1000, Hitachi, Tokyo, Japan) to enhance conductivity. Additionally, an energy dispersive spectrometer (EDS) was used to detect the elemental composition and distribution of the CINPs@MXene/PU/PDMS sponge.

### 2.4. Porosity Test of Sponge

The porosity of the sponges was determined by the displacement of liquids using ethanol in accordance with the methodologies reported. The volume of the sponges (V) was calculated, and a sample of known weight (W_0_) was immersed in ethanol for 1 h. Subsequently, the excess ethanol was removed, and the wet sponge was weighed (W_1_). The porosity was calculated using the following formula:

(1) $$Porosity=\left(W_{1}-W_{0}\right)/(\rho_{ethanol}\times V)]\times 100\%$$

### 2.5. Photothermal Performance and Stability Characterization of the Modified Sponge

To select the modified sponges with the best photothermal performance, the sponges (2 cm × 2 cm × 2 cm) were vertically irradiated using a 300 W xenon lamp to simulate solar illumination (at 1.0 sun, 1.0 kW·m^−2^) positioned 15 cm above the sponge surface, while an infrared thermal camera (FLIR E5, Wilsonville, OR, USA) was employed to record the real-time surface temperature variations of the material.

After selecting the sponge with the best photothermal performance, we conducted a test on the light absorption capacity of this modified sponge. The sponge samples, both before and after modification, were characterized using UV-vis diffuse reflectance spectroscopy (DRS) on a UV-vis spectrophotometer (Lambda 750 S, PerkinElmer, Shelton, CT, USA). The measurements were performed in the wavelength range of 200 to 800 nm at room temperature. BaSO_4_ was used as the reflectance standard.

To further evaluate the photothermal response characteristics and photothermal stability of the sponge, the modified sponge (2 cm × 2 cm × 2 cm) was vertically irradiated using a 300 W xenon lamp to simulate solar illumination (0.5 Sun, 1.0 Sun, 1.5 Sun, 0.5 kW·m^−2^, 1.0 kW·m^−2^, and 1.5 kW·m^−2^) positioned 15 cm above the sponge surface and the changes in surface temperature were recorded in real time by an infrared thermal imager. Meanwhile, five switching light cycles were adopted to test its photothermal stability at 1.0 Sun [23,24].

### 2.6. Hydrophobicity and Oleophilicity Test

To visually evaluate the surface wettability and oil affinity, qualitative hydrophobic/oleophilic experiments were conducted. Deionized water stained with methylene blue and oil stained with Sudan Red III were used as indicators. The interaction of these droplets with the experimental sponge and a control PU sponge was captured via time-lapse videography to analyze wettability behavior [25]. Additionally, the CINPs@MXene/PU/PDMS sponge was submerged in deionized water, and its response to immersion-induced wetting was documented for comparative evaluation [26,27].

To assess oil selectivity in an oil/water mixture, two representative oils were selected: light oil (peanut oil) and heavy oil (carbon tetrachloride), both of which were stained with Sudan Red III. The stained oils were carefully added to deionized water to form immiscible mixtures [26,28]. The CINPs@MXene/PU/PDMS sponge was then applied to absorb both types of oil from the water surface or bottom. The oil absorption process and separation behavior were recorded in real time using a phone camera for qualitative analysis.

### 2.7. Oil Absorption Capacity and Multiple Oil/Water Separation Efficiency Test

To evaluate the oil absorption capacity of the CINPs@MXene/PU/PDMS sponge, various oils and organic solvents, including peanut oil, vacuum pump oil, n-hexane, ethyl acetate, silicone oil, petroleum ether, xylene, chloroform, carbon tetrachloride, and dichloromethane, were used as test liquids. The sponge samples (2 cm × 2 cm × 2 cm) were weighed to obtain the initial mass (m_0_). Subsequently, the sponge was immersed in the target oil and organic solvent without external agitation for 5 min to ensure complete absorption of the oil and solvent. After removal, excess oil on the surface was gently blotted using filter paper, and the mass after oil uptake was recorded as m_1_. The oil absorption capacity was calculated using the following equation [29,30]:

(2) $$Absorption\;Capicity=\left(m_{1}-m_{0}\right)/m_{0}$$

Each test was repeated three times for statistical reliability.

To evaluate the oil absorption capacity of PU and CINPs@MXene/PU/PDMS sponges for light and heavy oils, 20 absorption cycles were performed. Post each absorption, the sponge was pressed to expel oil, dried with toilet paper, rinsed with ethanol, and dried again. The absorption capacity was calculated using Equation (2).

The oil/water separation performance was evaluated using immiscible mixtures of deionized water and oils, and organic solvents (dyed with Sudan Red III), including peanut oil, vacuum pump oil, n-hexane, ethyl acetate, silicone oil, petroleum ether, xylene, chloroform, carbon tetrachloride, and dichloromethane. Equal volumes (1:1, *v*/*v*) of water and oil were mixed to obtain a total volume of 10 mL. The CINPs@MXene/PU/PDMS sponge was gently placed in the mixture to absorb the oil phase selectively. After separation, the residual phase was collected and weighed (m_b_). The initial mass before separation was recorded as m_a_. The mass of the oil phase before separation is recorded as m_c_. The oil/water separation efficiency (η) was calculated as follows [31,32]:

(3) $$\eta ={{(m}_{a}-m}_{b})/m_{c}\times 100\%\;$$

All experiments were repeated three times, and the average efficiency was reported.

### 2.8. Continuous Oil/Water Separation Under Peristaltic Pump Driving

To conduct continuous oil/water separation experiments, the CINPs@MXene/PU/PDMS sponge was securely mounted at the silicone tube’s inlet, which was connected to a peristaltic pump. The test mixture, a stratified system with deionized water as the bottom layer and dyed peanut oil as the top layer, was pumped at a steady flow rate of 200 mL/min. Throughout the experiment, a smartphone camera was used to meticulously record the separation process. The formula for calculating oil flux was as follows:

(4) J=V/(A×t)

where J (L·m^−2^·h^−1^) is oil flux, V (L) is the volume of oil that has been completely absorbed and collected by the sponge or membrane, A (cm^2^) represents the cross-sectional area of the sponge in the separation device, and t (h) represents the time it takes to collect the oil with a volume of V.

### 2.9. Test of the Oil Absorption Capacity of Crude Oil

The vast majority of the large-scale and highly publicized crude oil spill water pollution incidents usually occur at sea [33]. Therefore, a simulated offshore oil spill was created to evaluate the crude oil absorption capacity of the modified sponge. Under simulated sunlight (1.0 Sun, 1.0·kW·m^−2^), 0.05 g of crude oil was dropped onto the surface of PU sponge and CINPs@MXene/PU/PDMS sponge [24]. The wetting of the crude oil drops was observed, and the corresponding wetting process was recorded. Additionally, the saturation absorption performance of CINPs@MXene/PU/PDMS sponge on crude oil under simulated sunlight conditions was also recorded. An amount of 5 g of crude oil was poured onto the surface of 20 g simulated seawater (the simulated seawater preparation was obtained by dissolving 23.0 g NaCl, 4.0 g Na_2_SO_4_, 11.0 g MgCl_2_, 0.6 g KCl, and 1.5 g CaCl_2_ in 1 L deionized water [34]). Under simulated sunlight provided by a 300 W xenon lamp (1 Sun, 1.0·kW·m^−2^), the CINPs@MXene/PU/PDMS sponge was used to absorb the crude oil (due to the high viscosity of crude oil, tweezers were used to gently assist the modified PU sponge in absorbing heavy oil). The absorption process was recorded in real time. The oil absorption capacity was calculated according to the following formula:

(5) $$il\;absorption\;rate=\left(W_{2}-W_{1}\right)/5\times 100\%$$

where W_2_ is the weight of the sponge after absorption, and W_1_ is the weight of the sponge before absorption.

To evaluate the saturation absorption capacity of the sponge for crude oil, we take the PU sponge and CINPs@MXene/PU/PDMS sponge with the specification of 1 cm × 1 cm × 1 cm, and then place them in the crude oil. We test under five different light conditions (no light, 0.5 sun, 1.0 sun, 1.5 sun, and 2.0 sun) and perform a 20-min light treatment separately. After the treatment, wipe off the residual crude oil on the sponge surface, weigh its mass, and calculate the saturation absorption amount of each sponge using Equation (2).

### 2.10. Cyclic Compression Performance Test

To evaluate the mechanical durability and structural stability of sponges in practical applications, a universal material testing machine (WDW-1000N, Chuanbai Instrument and Equipment Co., Ltd., Jinan, Shandong, China) was used to conduct compression tests on the sponges. During the test, the sample size was 2 cm × 2 cm × 2 cm. The compression rate was set at 10 mm/min, and the cyclic compression conditions of the sponge were recorded at the 1st, 100th, 200th, 300th, 400th, and 500th cycles under a strain of 80%. After each group of tests, the stress–strain curves were recorded [35].

Meanwhile, to evaluate the retention of surface functions after multiple mechanical deformations, the Water Contact Angle was measured to investigate the stability of their hydrophobicity.

### 2.11. Water Contact Angle Test

The Water Contact Angle (WCA) test, a widely recognized method for assessing surface wettability and hydrophobicity [36], was employed to measure the WCAs of the sponge material before and after surface modification using a contact angle goniometer (JCY-1, Fangrui Instrument Co., Ltd., Shanghai, China). To further evaluate the chemical stability of the CINPs@MXene/PU/PDMS sponge, the sponge (2 cm × 2 cm × 2 cm) was immersed in 50 mL of aqueous solutions with varying pH values (pH = 1–14). After 24 h of immersion, the sponges were removed and dried in an oven until they reached a constant weight. The static WCAs were then measured using a contact angle goniometer. To evaluate the abrasion resistance of the modified sponge, a sandpaper abrasion test was conducted using 400-grit sandpaper. The sponge sample was subjected to reciprocating abrasion over a distance of 20 cm. The test was performed for 5, 10, 20, 30, 40, and 50 cycles, respectively [37,38]. After each abrasion cycle, the WCA was measured to assess the retention of surface hydrophobicity. To simulate real-life application scenarios, the sponge samples were immersed in various natural water sources, including river water (Yangtze River, collected in Wuhan, Hubei Province), lake water (collected from East Lake, Tangxun Lake, Tang Lake, South Lake, Taizi Lake, and Houguan Lake in Wuhan, Hubei Province), and seawater (collected from the South China Sea, Sanya, Hainan Province). After soaking for 72 h, the samples were dried, and their static WCAs were measured to evaluate the stability of surface wettability. Thermal stability is a critical factor in assessing the performance of sponges in oil/water separation under elevated temperatures. To evaluate this property, the sponge samples were placed in an oven at different temperatures (60 °C, 80 °C, 100 °C, 120 °C, 140 °C, and 160 °C) for 24 h. After cooling to room temperature, the static WCAs were measured to examine the retention of surface hydrophobicity. Each sample was tested three times at different positions, and the average value was reported.

### 2.12. Flame-Retardant Performance Test

To systematically evaluate the mass change behavior and thermal stability of the materials during heat treatment, thermogravimetric analysis (TGA) was performed on both the unmodified PU sponge and the modified sponge. The experiment was conducted using a simultaneous thermal analyzer (STA449F3, NETZSCH, Waldkraiburg, Germany) under an air atmosphere, with a heating rate of 10 °C/min from room temperature to 500 °C. The thermal decomposition process and residual mass of the materials were recorded to compare their differences in thermal stability. To verify the sponge’s ability to suppress flames, we conducted flame-retardant tests on the sponge. A qualitative flame-retardancy test was performed to evaluate the combustion behavior of the sponge. Sponges (2 cm × 2 cm × 2 cm) were held vertically using metal tweezers and ignited with a butane lighter under ambient conditions. The process was recorded. Additionally, the presence or absence of molten or burning drips during combustion was carefully observed and documented [38,39,40].

### 2.13. Adsorption of Microplastics and Nanoplastics

To investigate the adsorption of the sponge for MPs, in this study, polystyrene (PS, 5 µm), polypropylene (PP, 100 µm), polyethene (PE, 150 µm), polyethene terephthalate (PET, 300 µm), and polymethyl methacrylate (PMMA, 1 mm) were chosen as the target pollutants for the adsorption experiment. The experimental procedure was as follows: a 1.0 mg/mL microplastic suspension was prepared via ultrasonication. For the adsorption experiments, 50 mg of the modified sponge was added to 50 mL of this suspension and agitated at 200 rpm for 8 h. After reaching equilibrium, the sponge was removed, dried, and weighed. The experiments were performed at room temperature (25 °C). The adsorption rate of microplastics can be calculated using the following formula [41,42]:

(6) $$adsorption\;ability\;(Q)=1000\times {(m}_{1}-m_{0})/m_{0}$$

where Q (mg/g) is the amount of adsorbed microplastic per unit mass of sponge, and m_0_ and m_1_ (g) are the weight of the sponge before and after adsorption, respectively.

For the adsorption of nanoplastics (NPs), a series of batch adsorption experiments was conducted using 100 nm PS NPs as the model pollutant. The experiments were performed at room temperature (25 °C). Specifically, 50 mg of the sponge adsorbent was added to 50 mL of a PS NPs suspension with a concentration of 200 mg/L. The mixture was then agitated at 200 rpm. At predetermined time intervals (2, 4, 6, 8, 10, 12, 16, 20, and 24 h), an aliquot of the suspension was withdrawn. The fluorescence intensity of the supernatant was measured using a fluorescence spectrophotometer (F-7000, Hitachi, Japan), and the residual concentration of PS NPs in the supernatant was determined based on a pre-established calibration curve. The adsorption capacity at time t (Q_t_, mg/g) was calculated using the following equation:

(7) $$Q_{e}={(C}_{0}-C_{e})\times V/m$$

(8) $$Q_{t}=\left(C_{0}-C_{t}\right)\times V/m$$

where C_0_ (mg/L) and C_e_ (mg/L) represent the initial and equilibrium concentrations of the fluorescent PS NPs, respectively. C_t_ (mg/L) is the concentration of the fluorescent PS NPs in the solution at a given time point. Q_e_ (mg/g) and Q_t_ (mg/g) represent the adsorption capacity upon equilibrium and at a given time point, respectively. The mass of sponge and reaction volume are cited as m (g) and V (L), respectively.

To elucidate the adsorption kinetics and underlying mechanisms of PS NPs onto Fe_3_O_4_@MXene-OA, the adsorption capacity as a function of contact time was first investigated. To quantitatively describe the adsorption rate and explore the potential rate-controlling steps, four classic kinetic models were employed to fit the experimental data: the pseudo-first-order (PFO, Equation (9)), pseudo-second-order (PSO, Equation (10)), Elovich (Equation (11)), and Weber–Morris (W–M) models (Equation (12)).

(9) $$\log\left(Q_{e}-Q_{t}\right)=logQ_{e}-\frac{k_{1}}{2.303}t$$

(10) $$\frac{t}{Q_{t}}=\frac{1}{k_{2}\times Q_{e}^{2}}\times \frac{1}{Q_{e}}t$$

(11) $$Q_{t}=\frac{1}{\beta }ln(1+k_{e}\beta t)$$

(12) $$Q_{t}=k_{w}t^{0.5}+C$$

To evaluate the maximum adsorption capacity and the surface interaction behavior in equilibrium, an adsorption isotherm study was carried out. The Langmuir isotherm model (Equation (13)) and Freundlich isotherm model (Equation (14)) were used to analyze the equilibrium adsorption data.

(13) $$=\frac{1}{K_{l}Q_{m}}+\frac{C_{e}}{Q_{m}}$$

(14) $$Q_{e}=K_{f}C_{e}^{1/n}$$

The thermodynamic characteristics of the adsorption process were investigated by evaluating the adsorption performance at different temperatures of 25 °C, 35 °C, and 45 °C. All these experiments lasted for 20 h, and the initial PS NPs concentration was set at 200 mg/L.

We conducted saturation adsorption experiments for PS NPs of different concentrations. We chose 5 mg/L, 10 mg/L, 25 mg/L, 50 mg/L, 100 mg/L, 150 mg/L, and 200 mg/L PS NPs, and the adsorption experiment was conducted for 24 h.

### 2.14. Influence of Typical Factors on Adsorption Performance

To evaluate the applicability of CINPs@MXene/PU/PDMS sponge in complex environments, we investigated the influence of coexisting substances on their adsorption performance. These coexisting substances included silica nanoparticles (SiO_2_), lead ions (heavy metal), methylene blue (organic dye), and ethanol (other solvents). By mixing 100 nm PS NPs with the above substances and oscillating them on an oscillator for 48 h, PS-SiO_2_, PS-lead ions, PS-methylene blue, and PS-ethanol coexisting suspensions/solutions were prepared to simulate the actual aquatic environment. The concentrations of silica, lead ions, methylene blue, and ethanol were 1 mg/mL, 25 mg/L, 0.5 mg/L, and 50% (*v*/*v*), respectively. Subsequently, approximately 5 mg of CINPs@MXene/PU/PDMS sponge was added to each 5 mL coexisting system. The adsorption capacity of the sponges was calculated by measuring the fluorescence intensity of the systems before and after adsorption. The standard curve solutions of all systems were prepared separately, and the standard solutions were treated accordingly based on the types of coexisting substances.

### 2.15. Evaluation of Adsorption Performance in the Actual Water Environment

To evaluate the practical applicability of the modified sponge in complex practical environments, the efficiency of removing PS NPs in four representative water samples was evaluated. These four water samples are (i) Yangtze River water in Wuhan, China; (ii) China South Sea collected in Yazhou District, Sanya, China; (iii) East Lake of Wuhan, China; (iv) a typical takeout soup, that was, “Yang Guofu Spicy Hot Pot”. Fluorescent PS NPs were added to these four water samples, respectively, and the final concentration reached 200 mg/L. The final concentration of PS NPs was determined by the above-mentioned fluorescence method, and the removal efficiency was calculated by Equation (7). In addition, the reusability of modified sponges was evaluated through five consecutive adsorption–desorption cycles. After each adsorption experiment, the sponge was removed, loaded with PS NPs, and gently squeezed to remove excess liquid. The regeneration process is to immerse the sponge in a beaker with ethanol and treat it with ultrasound for 30 min. Then, wash the regenerated sponge with ethanol and dry it in a 60 °C oven for the next adsorption cycle.

## 3. Results

### 3.1. Structural and Compositional Characterization of CINPs, MXene, CINPs@MXene, and Sponges

**Figure 3:**
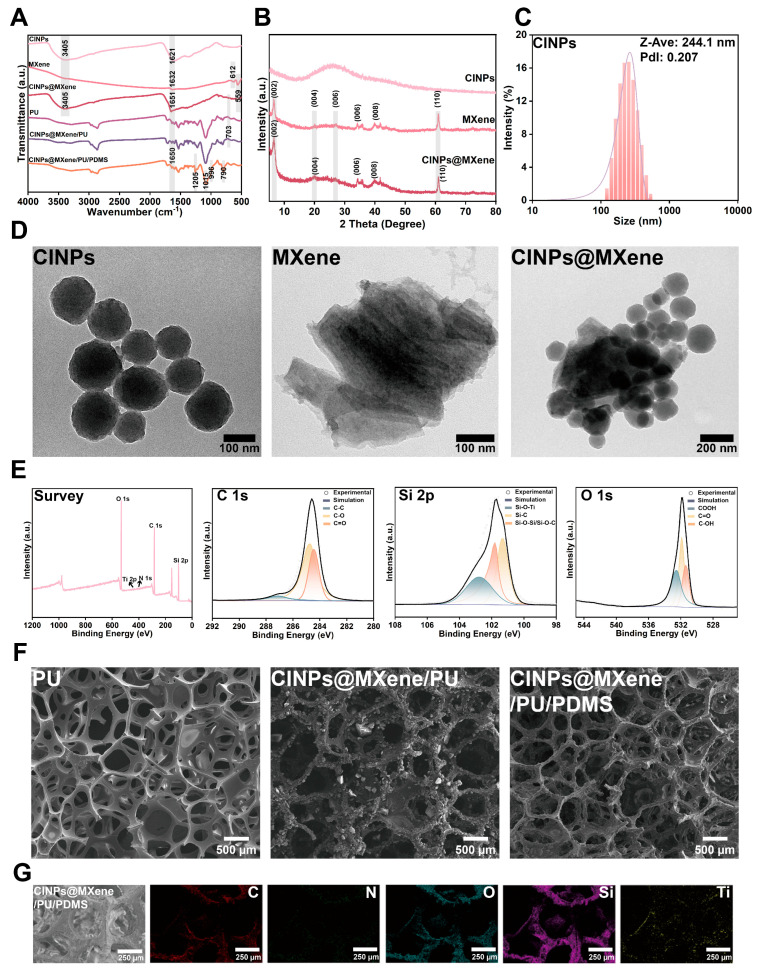
Spectroscopic and structural verification of the step-by-step synthesis of the modified sponge. (**A**) FT-IR spectra of CINPs, MXene, CINPs@MXene, PU, CINPs@MXene/PU sponge, and CINPs@MXene/PU/PDMS sponge. (**B**) XRD of CINPs, MXene, and CINPs@MXene. (**C**) Hydrated particle size of CINPs. (**D**) TEM images of CINPs, MXene, and CINPs@MXene. (**E**) XPS survey spectrum of the CINPs@MXene/PU/PDMS sponge. (**F**) SEM images of PU sponge, CINPs@MXene/PU sponge, and CINPs@MXene/PU/PDMS sponge. (**G**) EDS elemental mapping images of CINPs@MXene/PU/PDMS sponge.

The FT-IR spectra in Figure 3A offer compelling evidence of the successful synthesis and integration of CINPs, MXene, and PU/PDMS into various composites. CINPs display distinctive peaks at 3405 cm^−1^ (O–H/N–H) and 1621 cm^−1^ (C=O or aromatic C=C), matching melanin-like materials [43]. MXene’s spectrum reveals peaks at 1632 cm^−1^ (–OH bending/C=O stretching), 612 cm^−1^ (Ti–C), and 559 cm^−1^ (Ti–O), confirming its layered metal carbide structure with surface oxides [43]. The CINPs@MXene composite’s spectrum combines these features, indicating molecular-level integration.

In the CINPs@MXene/PU sponge, CINPs’ characteristic peak at 1650 cm^−1^ and MXene’s peak around 703 cm^−1^ confirm their presence in the PU matrix. The CINPs@MXene/PU/PDMS sponge’s spectrum adds PDMS-specific peaks at 1250 cm^−1^ (Si–CH_3_ bending), 1015 cm^−1^, 996 cm^−1^ (Si–O–Si asymmetric stretching), and 790 cm^−1^ (Si–CH_3_/Si–O–Si rocking), verifying PDMS coating and siloxane structures on the material surface. These results collectively validate the effective incorporation of each component into the composites, supporting their potential multifunctional applications.

Moreover, the XRD pattern of CINPs (Figure 3B) shows no distinct crystalline peaks, indicating that the material is amorphous, consistent with the non-crystalline characteristics of melanin-like materials. The XRD pattern of MXene displays a series of characteristic diffraction peaks, further confirming its layered structure and good crystallinity. As shown in the figure, a prominent (002) diffraction peak appears at a low 2θ angle, indicating the typical layered stacking structure of MXene. The presence, position, and intensity of this peak reflect the interlayer spacing and are regarded as a distinguishing feature of MXene materials. In addition, higher-order diffraction peaks corresponding to the (004), (006), and (008) planes can be observed at higher angles. These peaks are harmonics of the (002) reflection and further demonstrate the periodic layered structure of MXene, indicating a well-ordered stacking of nanosheets. Notably, a diffraction peak corresponding to the (110) plane is also observed around 38°, which reflects the interatomic spacing of Ti planes in MXene. The presence of this peak suggests that the Ti–C framework of the MXene structure remains intact even after the removal of some elements (such as Al), confirming the structural integrity of the Ti–C lattice. For the CINPs@MXene composite material, the XRD pattern displays the characteristic diffraction peaks of MXene, including those of the (002), (004), (006), (008), and (110) planes. This indicates that the layered crystal structure of MXene is preserved during the formation of the composite with CINPs. It suggests that the introduction of CINPs does not disrupt the ordered layered structure of MXene, and that the composite is likely formed through non-destructive physical adsorption or mild chemical interactions, providing a structural basis for subsequent performance enhancements [44].

The particle size distribution of CINPs is depicted in Figure 3C, revealing a Z-average particle size of 244.1 nm and a PdI of 0.207. This indicates a narrow size distribution and high uniformity, which are vital for the nanoparticles’ dispersibility and colloidal stability. These properties are essential for their effective integration with MXene and the enhancement of multifunctional properties in composite applications.

The TEM images in Figure 3D reveal the distinct morphologies of CINPs, MXene, and their composite. CINPs display a uniform spherical shape with an average size of 126.19 ± 25.65 nm, as quantified by Image J2 (see Figure S1). Their size is notably smaller than when hydrated, likely due to aggregation in aqueous settings. MXene, on the other hand, presents a layered structure with neatly stacked nanosheets and crisp edges. In the CINPs@MXene, CINPs are evenly distributed across the MXene sheets’ surfaces, edges, and interlayers. Importantly, this combination does not disrupt MXene’s layered architecture, demonstrating its stability during composite creation.

Figure 3E presents the XPS survey spectrum of the CINPs@MXene/PU/PDMS sponge, revealing distinct peaks for C 1s, O 1s, and Si 2p, which confirm the integration of carbon, oxygen, and silicon elements into the composite. The detection of Ti and N signals further evidences the successful incorporation of MXene and CINPs. The high-resolution C 1s spectrum exhibits peaks corresponding to C–C, C–O and C=O bonds, indicating a surface enriched with reactive oxygen-containing groups. The Si 2p spectrum shows peaks attributed to Si–C, Si–O–Ti, and Si–O–Si/Si–O–C bonds, suggesting stable interactions between PDMS and MXene/CINPs. The O 1s spectrum identifies COOH, C=O, and C–OH groups, highlighting the presence of polar functional groups that enhance surface activity and potentially contribute to the material’s functionalities. These findings collectively underscore the composite’s structural stability and multifunctional potential.

The hydrophobic modification of the PU sponge was achieved through two approaches: increasing microscale roughness with nanostructured coatings to trap air, and decreasing surface energy by incorporating PDMS to enhance water repellency. As shown in Figure 3F, the PU sponge exhibits a smooth skeleton structure with moderately distributed pores. In contrast, the PU sponge modified with CINPs@MXene exhibits a significantly rougher surface morphology, characterized by irregular micro- and nano-scale textures. Notably, no excess CINPs@MXene aggregates were observed within the pores, indicating strong interfacial compatibility and uniform adhesion of CINPs@MXene onto the PU skeleton. Furthermore, in the CINPs@MXene/PU/PDMS sponge, a continuous and well-defined PDMS coating was observed enveloping the surface. This coating not only fully encapsulates the CINPs@MXene/PU skeleton but also preserves the underlying rough surface structure, effectively forming a hierarchical surface topology. The EDS mapping of the CINPs@MXene/PU/PDMS sponge reveals a homogeneous distribution of the primary elements (C, N, O, Si, and Ti) across the sponge skeleton. As shown in Figure 3G and Figure S2, and Table S1, the presence of N (from PU and CINPs) and Ti (from MXene) confirms the successful incorporation of CINPs and MXene into the sponge framework. This observation aligns well with the XPS results, which further validate the chemical composition and surface functionalization of the composite.

### 3.2. Evaluation of the Morphology, Optical Properties, and Photothermal Properties of CINPs@MXene/PU/PDMS Sponge

**Figure 4:**
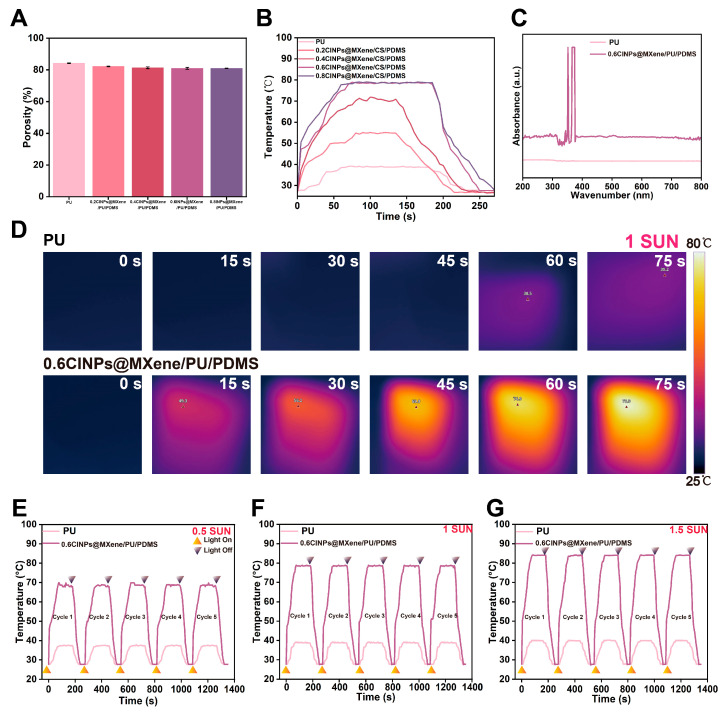
Morphological, optical, and photothermal characterization of CINPs@MXene/PU/PDMS sponge. (**A**) Porosity of pure PU sponge and modified sponges with various CINPs@MXene doping concentrations. (**B**) Photothermal heating and cooling curves of pure PU sponge and modified sponges with different CINPs@MXene doping concentrations under 1.0 Sun illumination. (**C**) UV-vis absorption spectra of pure PU sponge and 0.6CINPs@MXene/PU/PDMS sponge. (**D**) Infrared thermal images of pure PU sponge and 0.6CINPs@MXene/PU/PDMS sponge under 1 Sun illumination at different time points. (**E**–**G**) Photothermal heating-cooling cycling curves of pure PU sponge and 0.6CINPs@MXene/PU/PDMS sponge for five cycles under 0.5 Sun (**E**), 1.0 Sun (**F**), and 1.5 Sun (**G**) light intensities.

Figure 4A shows that all sponges maintain a high porosity of more than 80%. With the increase in the load of CINPs@MXene, the porosity of the modified sponge decreased slightly, but the change was not significant. This shows that the CINPs@MXene has successfully retained the inherent porous structure characteristics of PU sponge, which provides favorable conditions for adsorption applications.

Figure 4B shows that the photothermal response of pure PU sponges was weak, while all CINPs@MXene/CS/PDMS sponges show significant photothermal effects. With the increase in the load of CINPs@MXene, the heating rate of the composite sponge was significantly improved. Among them, the modified sponges with a load of 0.6 wt% and 0.8 wt% can quickly reach the maximum temperature of about 80 °C and cool down quickly after the light stops, indicating that they have efficient and stable photothermal conversion performance. The comparable photothermal conversion efficiencies observed for the sponges with 0.6 wt% and 0.8 wt% CINPs@MXene loadings can be attributed to the saturation of light absorption and the self-shielding effect. For the 0.6CINPs@MXene/PU/PDMS sponge, the skeleton surface is already densely covered by the photothermal components, enabling the capture of the vast majority of incident light. Further increasing the loading to 0.8 wt% leads to the excessive stacking of nanoparticles. Consequently, the inner layers of the photothermal agents are shielded from light irradiation and contribute negligibly to additional heat generation, resulting in a performance plateau. Based on this, the subsequent experiments were carried out by selecting modified sponges with CINPs@MXene doping of 0.6 wt%.

The light absorption performance of the PU sponge before and after modification was evaluated using UV-vis absorption spectra. As shown in Figure 4C, the pristine PU sponge exhibited weak absorption across the 200–800 nm wavelength range, with a nearly flat spectral profile. In contrast, the 0.6CINPs@MXene/PU/PDMS sponge displayed significantly enhanced absorption, particularly at 352 nm and in the range of 365–377 nm. This improvement indicates that the incorporation of CINPs and MXene enhanced the sponge’s light-harvesting capability, making it suitable for photothermal conversion.

The photo-responsive performance of both sponges was assessed under different light intensities (0.5 Sun, 1.0 Sun, and 1.5 Sun) using a 300 W xenon lamp as the light source. Real-time monitoring of surface temperatures was carried out with the infrared thermal camera. Under 1.0 Sun illumination, the PU sponge showed minimal temperature increase, reaching only 39.1 °C after 60 s, while the 0.6CINPs@MXene/PU/PDMS sponge rapidly heated up to 49.3 °C within 15 s and continued to rise, reaching 78.9 °C after 75 s. This demonstrates the significantly enhanced photothermal responsiveness of the modified sponge. Under all light intensities, the modified sponge exhibited rapid temperature increases compared to the PU sponge. Under 0.5 Sun, the modified sponge reached a maximum temperature of 69.9 °C within 75 s, whereas the PU sponge only reached 37 °C. Under 1.5 Sun, the modified sponge reached 84.1 °C, while the PU sponge only slightly increased to 40 °C. In all cases, the modified sponge showed rapid cooling once the light source was turned off, indicating efficient thermal response and dynamic thermal regulation. The photothermal stability of the 0.6CINPs@MXene/PU/PDMS sponge was also tested over five consecutive heating–cooling cycles under varying light intensities. The sponge maintained consistent maximum temperatures and heating rates across cycles, confirming its excellent durability and stability in photothermal applications.

### 3.3. Wettability, Selective Oil Absorption, and Mechanical Performance of CINPs@MXene/PU/PDMS Sponge

**Figure 5:**
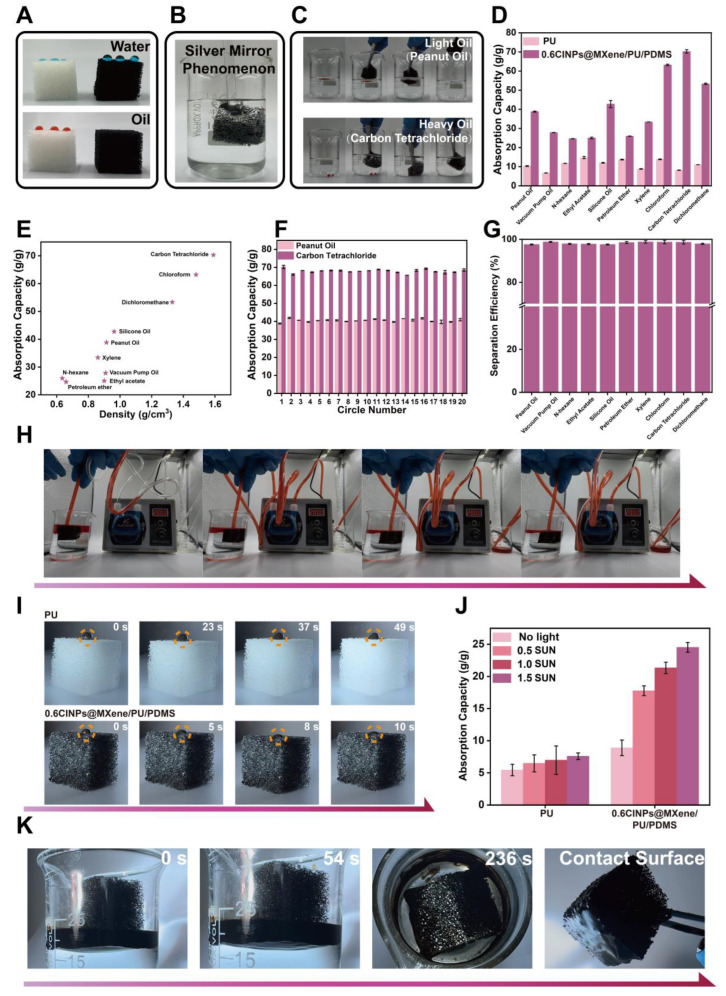
Oil–water separation performance and absorption ability of the 0.6CINPs@MXene/PU/PDMS sponge. (**A**) Wettability tests of the PU sponge and the 0.6CINPs@MXene/PU/PDMS sponge with water (top) and oil (bottom) droplets. (**B**) A photograph of 0.6CINPs@MXene/PU/PDMS sponge showing a “silver mirror phenomenon” when submerged in water. (**C**) The removal process of a light oil (peanut oil) and a heavy oil (carbon tetrachloride) from water by 0.6CINPs@MXene/PU/PDMS sponge. (**D**) Absorption capacities of the PU sponge and 0.6CINPs@MXene/PU/PDMS sponge for various oils and organic solvents. (**E**) The relationship between the absorption capacity of 0.6CINPs@MXene/PU/PDMS and the density of the absorbed liquids. (**F**) Absorption capacity of 0.6CINPs@MXene/PU/PDMS sponge for peanut oil and carbon tetrachloride over 20 cycles. (**G**) The separation efficiency of 0.6CINPs@MXene/PU/PDMS sponge for various oil–water mixtures. (**H**) A demonstration of the continuous oil–water separation setup using 0.6CINPs@MXene/PU/PDMS sponge and a peristaltic pump. (**I**) Time-lapse images of the crude oil absorption process on the PU sponge and 0.6CINPs@MXene/PU/PDMS sponge. (**J**) Absorption capacity of the two sponges for crude oil under different light intensities. (**K**) The process of light-driven absorption and collection of viscous crude oil from 0.6CINPs@MXene/PU/PDMS sponge.

Figure 5A demonstrates the selective absorption capacity of the PU sponge before and after modification towards water and oil. In the experiments, deionized water and peanut oil were dyed with methylene blue and Sudan red III, respectively. The results indicate that upon water contact, the pristine PU sponge exhibits specific hydrophilic absorption capacity, whereas the 0.6CINPs@MXene/PU/PDMS sponge shows pronounced hydrophobic characteristics. For oil absorption, the unmodified PU sponge displays negligible absorption capability, while the 0.6CINPs@MXene/PU/PDMS sponge demonstrates rapid and repeatable oil absorption performance. Furthermore, Supplementary Video S1 indicates that when a water droplet is deposited on the surface of the 0.6CINPs@MXene/PU/PDMS sponge, it remains intact without being absorbed and rapidly rolls off. This observation further confirms the pronounced hydrophobic nature of the CINPs@MXene/PU/PDMS sponge.

To further validate the hydrophobic properties of the sponge, we performed an immersion test by applying an external force to submerge the 0.6CINPs@MXene/PU/PDMS sponge underwater. Figure 5B revealed the formation of a distinct “silver mirror effect”—a trapped air layer on the sponge surface—effectively preventing water infiltration. This phenomenon provides additional evidence of the material’s robust hydrophobicity [31].

The oil/water separation performance of the 0.6CINPs@MXene/PU/PDMS sponge was assessed using light oil (peanut oil) and dense oil (carbon tetrachloride) as model contaminants. As demonstrated in Figure 5C and supported by Videos S2 and S3, the modified sponge exhibited rapid and efficient absorption of both oil types. These results indicate the material’s excellent absorption capacity for various oils and organic solvents. We subsequently conducted saturated absorption experiments using both pristine PU sponge and 0.6CINPs@MXene/PU/PDMS sponge with multiple oils and organic solvents. As shown in Figure 5D, the results demonstrate that the modified sponge exhibits significantly enhanced absorption capacity for all tested oils and organic compounds compared to the unmodified sponge. The absorption capacity tests revealed that the modified PU sponge exhibited considerably improved performance compared to the pristine PU sponge, with absorption multiples ranging from 1.69-fold for ethyl acetate to 8.57-fold for carbon tetrachloride, demonstrating its superior affinity for a diverse range of organic compounds. Furthermore, the saturation absorption capacity of this material for various oils and organic solvents is correlated with the density of the liquid, as shown in Figure 5E. The cyclic absorption tests (Figure 5F) demonstrated excellent stability of the 0.6CINPs@MXene/PU/PDMS sponge, maintaining absorption capacities of 38.81–41.91 g/g for peanut oil and 66.05–70.28 g/g for carbon tetrachloride over 20 consecutive cycles, with coefficients of variation as low as 3.8% and 3.1% respectively, confirming the material’s outstanding reusability and structural integrity for both light and dense oils.

The sponge’s oil/water separation efficiency was further evaluated, specifically its capacity to handle diverse oils and solvents. Experimental results (Figure 5G) demonstrate that the 0.6CINPs@MXene/PU/PDMS sponge exhibits an extremely high oil/water separation capacity for different types of oils and organic solutions. Whether dealing with common mineral oils, vegetable oils, or more complex organic solvents, the separation efficiency of the sponge remains above 97%, showing excellent separation performance. This result not only confirms the high efficiency of the sponge in processing various oil/water mixtures but also indicates its ability to meet the demands of oil/water separation in a variety of complex environments.

In a continuous oil absorption test, the 0.6CINPs@MXene/PU/PDMS sponge was subjected to constant suction from a peristaltic pump. As depicted in Figure 5H, the sponge exhibited stable and efficient oil uptake and transport, underscoring its potential for real-world applications. It has been calculated that the oil flux of the modified sponge was 22542 L⋅m^−2^⋅h^−1^.

The crude oil absorption performance of the sponges was evaluated under 1.0 Sun illumination. When crude oil was placed on the PU sponge, it remained on the surface without being absorbed, as the reduction in oil viscosity under simulated sunlight was insufficient to enable absorption. In contrast, the 0.6CINPs@MXene/PU/PDMS sponge demonstrated rapid oil adsorption, absorbing crude oil within 10 s, highlighting its effective photothermal-assisted adsorption properties.

Figure 5J clearly shows the difference in the saturation absorption performance of 0.6CINPs@MXene/PU/PDMS sponge on crude oil under different light intensities compared with pure PU sponge. It can be seen that the absorption capacity of the modified sponge far exceeds that of pure PU sponge under all conditions, and its performance was significantly positively correlated with the light intensity. As the light was enhanced from no light to 1.5 sun, its saturation absorption has jumped from about 9 g/g to nearly 25 g/g. In contrast, the absorption capacity of pure PU sponge was not only low in base, but also was almost not affected by light intensity. This result shows that 0.6CINPs@MXene/PU/PDMS sponge is a high-efficiency light-responsive absorption material with great application potential in the use of solar energy for oil spill control and other fields.

Further testing revealed that the modified sponge absorbed 4.57 g of crude oil, or approximately 91.4% of the total 5 g, within 4 min of illumination. The unabsorbed oil primarily adhered to the beaker walls. These results all demonstrate the strong absorption capacity of the modified sponge for crude oil, making it an excellent candidate for oil/water separation and related applications.

### 3.4. Durability and Environmental Stability of CINPs@MXene/PU/PDMS Sponge

**Figure 6:**
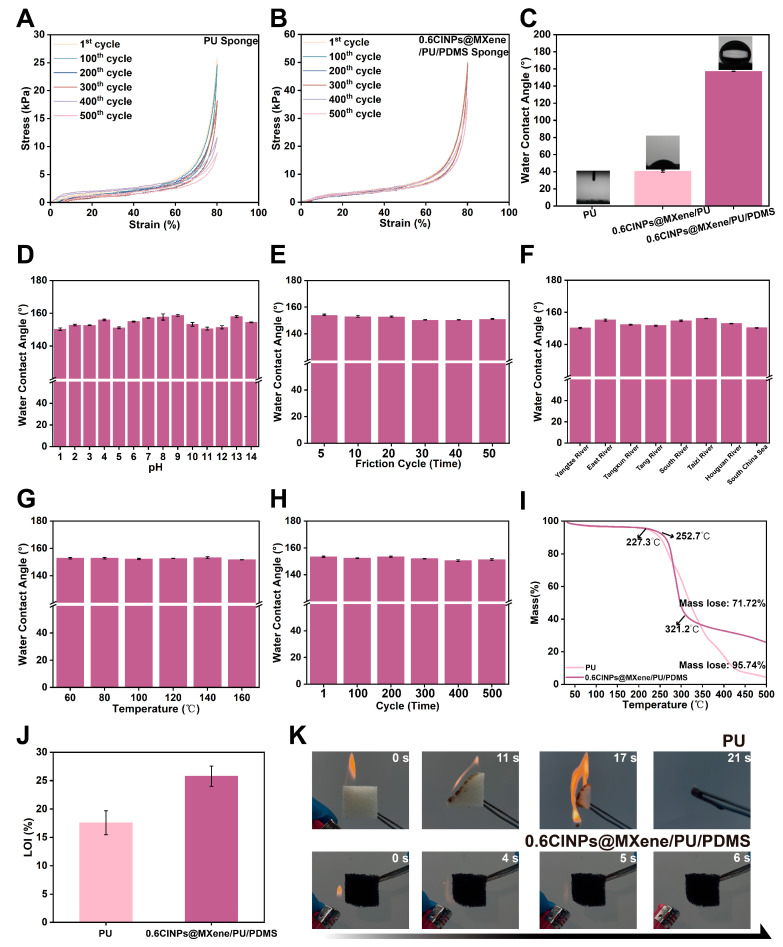
Mechanical properties, chemical stability, and flame retardancy of the 0.6CINPs@MXene/PU/PDMS sponge. (**A**,**B**) Compressive stress–strain curves of the (**A**) PU sponge and (**B**) 0.6CINPs@MXene/PU/PDMS sponge over 500 cycles. (**C**) WCAs of PU sponge, 0.6CINPs@MXene/PU sponge, and 0.6CINPs@MXene/PU/PDMS sponge. (**D**) WCAs of 0.6CINPs@MXene/PU/PDMS sponge after immersion in solutions with different pH values. (**E**) WCAs of 0.6CINPs@MXene/PU/PDMS sponge after various abrasion cycles. (**F**) WCAs of 0.6CINPs@MXene/PU/PDMS sponge after immersion in different water environments. (**G**) WCAs of 0.6CINPs@MXene/PU/PDMS sponge after thermal treatments at different temperatures. (**H**) WCAs of 0.6CINPs@MXene/PU/PDMS sponge after various compression cycles. (**I**) TGA curves of the PU sponge and 0.6CINPs@MXene/PU/PDMS sponge. (**J**) The LOI test results of the PU sponge and 0.6CINPs@MXene/PU/PDMS sponge. (**K**) Photographs from the combustion tests of the PU and 0.6CINPs@MXene/PU/PDMS sponge.

The cyclic compressive performance of the sponge, both before and after modification, was evaluated to assess its reusability. As shown in Figure 6A,B, the PU sponge exhibited a maximum stress of 25.667 kPa during the first compression at 80% strain. However, with increasing cycle numbers, the maximum stress gradually decreased, reaching 8.778 kPa after 500 cycles, indicating significant fatigue behavior. In contrast, the 0.6CINPs@MXene/PU/PDMS sponge showed a significantly enhanced mechanical performance, with a maximum stress of 52.797 kPa under the same compression conditions, approximately 2.06 times higher than that of the pristine PU sponge. After 300 compression cycles, the stress only slightly decreased to 50.685 kPa, representing a reduction of less than 4%. Even after 400 and 500 cycles, the maximum stresses remained at 37.131 kPa and 36.869 kPa, respectively. Despite some decline, the modified sponge retained substantially better mechanical strength than the unmodified one, demonstrating excellent structural durability and resilience during repeated compressive cycles, thus offering promising potential for practical applications.

Figure 6C illustrates the WCAs of the PU sponge at different modification stages. Initially, the water droplet was immediately absorbed by the pristine PU sponge (WCA ≈ 0°). After loading with CINPs@MXene, the WCA increased slightly to 40.68°. This indicates that while the incorporation of CINPs@MXene significantly enhanced the surface roughness, the inherent hydrophilicity of the nanoparticles prevented the surface from becoming hydrophobic. To further elucidate the synergistic mechanism, a control experiment was conducted on a PU sponge coated only with PDMS (PU/PDMS). As shown in Figure S3, the PU/PDMS sponge exhibited a WCA of 130.8°, which corresponds to a hydrophobic but not superhydrophobic state. This suggests that the low surface energy of PDMS alone is insufficient to achieve superhydrophobicity without adequate surface roughness. However, for the final CINPs@MXene/PU/PDMS sponge, the WCA rose significantly to 157.17°. This dramatic enhancement confirms that the superhydrophobicity is the result of a synergistic effect: the CINPs@MXene layer constructs the essential micro/nano-hierarchical roughness, while the PDMS coating provides the necessary low surface energy.

The sponge’s performance was then evaluated in terms of key stability metrics essential for practical applications, including chemical stability, friction durability, thermal stability, and compressive strength. These properties directly affect the sponge’s functionality in oil/water separation tasks under various environmental conditions. The chemical stability test, conducted by immersing the sponge in aqueous solutions of varying pH for 24 h, showed that the WCA remained high across both acidic and alkaline conditions. Only minor reductions in WCA were observed under extreme pH values, indicating that the sponge can maintain its hydrophobicity in a wide range of pH environments. The friction durability was assessed through repeated cycles, revealing that after 20 cycles, the WCA showed negligible change. Even after 30–50 cycles, the WCA remained above 150°, demonstrating excellent wear resistance. To assess stability in aquatic environments, the sponge was immersed in water from the Yangtze River, several lakes in Wuhan, and seawater from the South China Sea for 72 h. The WCA remained above 150° in all water bodies, indicating good stability in diverse aquatic environments. In terms of thermal stability, exposure to temperatures up to 160 °C did not significantly alter the WCA, confirming that the sponge retains its superhydrophobic properties even under elevated temperatures. Finally, the compressive durability was evaluated by measuring the WCA after multiple compression cycles. The results showed that the sponge maintained a WCA above 150° after several compression cycles, highlighting its exceptional compressive strength and reusability. These results suggest that the CINPs@MXene/PU/PDMS sponge exhibits remarkable stability across multiple performance metrics, ensuring reliable and long-term efficiency in demanding oil–water separation applications.

In practical applications, oil/water separation materials are required not only to exhibit excellent separation efficiency and absorption capacity, but also to possess good thermal stability and flame retardancy to withstand complex and variable operating environments. In this study, beyond the functional evaluation of the 0.6CINPs@MXene/PU/PDMS sponge, TGA analysis and flame-retardant tests were conducted to comprehensively assess the material’s stability and safety under high-temperature and open-flame conditions [45]. Thermal stability testing showed in Figure 6I that both the pristine PU sponge and the modified 0.6CINPs@MXene/PU/PDMS sponge exhibited minimal weight loss (around 3.7%) up to 227.3 °C. However, the PU sponge exhibited rapid degradation at higher temperatures, with a total weight loss of 95.74% by 500 °C. In contrast, the 0.6CINPs@MXene/PU/PDMS sponge showed enhanced thermal stability, with a weight loss of only 71.72% at 500 °C, indicating better structural integrity under high temperatures.

The LOI is a key indicator to measure the difficulty of combustion of materials. The higher its value, the better the flame-retardant performance of the material. It can be clearly seen from Figure 6J that the LOI value of 0.6CINPs@MXene/PU/PDMS sponge (about 26%) was significantly higher than that of pure PU sponge (about 17.5%). This shows that through composite modification, the flame-retardant performance of the sponge has been greatly improved, making it less prone to combustion and higher safety in practical applications.

Finally, the combustion behavior of both sponges was tested under direct flame exposure. As shown in Figure 6K, the pristine PU sponge ignited within 3 s of flame contact, burning rapidly with visible molten droplets, and was completely consumed in 21 s. In contrast, the modified sponge demonstrated significantly improved flame resistance. Upon flame contact, it only exhibited slight surface shrinkage, the release of a few black particles, and brief sparks, but self-extinguished once the flame was removed, showing no sustained combustion. This self-extinguishing behavior highlights the enhanced flame-retardant properties of the 0.6CINPs@MXene/PU/PDMS sponge, making it suitable for applications in high-temperature or flammable environments. The superior flame retardancy is attributed to a synergistic protection mechanism between the coating and the functional filler. First, the outer PDMS layer undergoes in situ ceramization upon exposure to flame, forming a thermally stable silica (SiO_2_) barrier that limits heat transfer [46]. Simultaneously, the incorporated MXene nanosheets not only act as a physical barrier to impede the diffusion of oxygen and volatile decomposition products but also undergo thermal oxidation to form a titanium dioxide (TiO_2_) ceramic layer [47]. This combined SiO_2_/TiO_2_ ceramic shield effectively reinforces the char layer, suppressing flame propagation and preventing melt-dripping.

### 3.5. Evaluation of Microplastic Adsorption of the Modified Sponge

**Figure 7:**
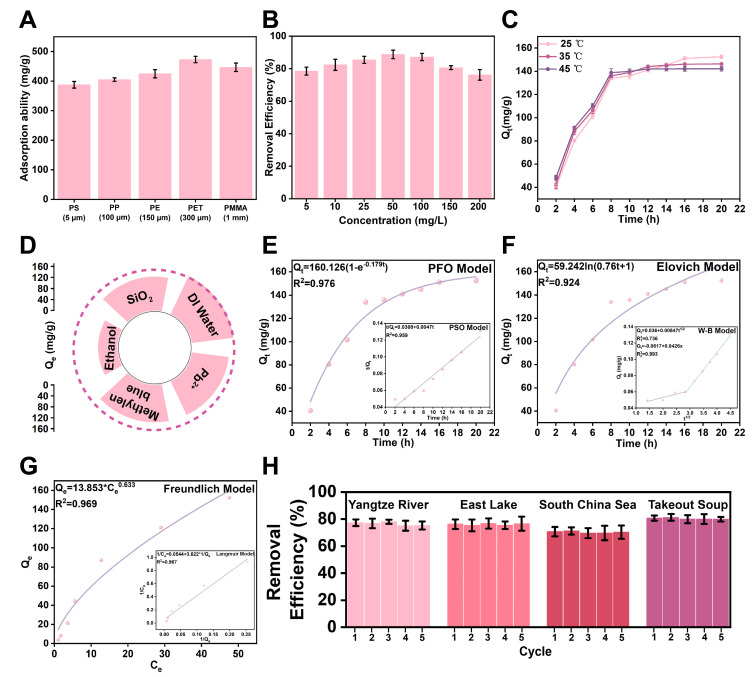
Study on the adsorption performance, mechanism and application potential of the modified sponge for microplastics. (**A**) Adsorption capacity for various types of microplastics. (**B**) Effect of initial concentration on removal efficiency. (**C**) Effect of temperature on adsorption kinetics. (**D**) Adsorption selectivity test. (**E**) Pseudo-first-order and pseudo-second-order kinetic model fitting. (**F**) Elovich and Weber–Morris model fitting. (**G**) Freundlich and Langmuir isotherm model fitting. (**H**) Cycling performance test in real water samples.

Figure 7A shows the adsorption capacity of the modified sponge on five common microplastics (PS, PP, PE, PET, PMMA). The results show that the sponge showed an adsorption capacity of up to 400 mg/g or even higher on all tested microplastics, among which the adsorption capacity for PET was the strongest. This proves that the modified sponge is a broad-spectrum and efficient microplastic adsorbent.

Figure 7B studies the effect of the initial concentration of PS NPs on the efficiency of sponge removal. Good adsorption efficiency was maintained in the wide concentration range of 5 to 200 mg/L, and reached a peak in the concentration range of 50–100 mg/L, close to 90%. This shows that the material has stable and excellent performance in dealing with microplastic pollution at different concentration levels.

Figure 7C compares the effects of different temperatures (25, 35, 45 °C) on adsorption kinetics. The results show that the adsorption equilibrium can be basically achieved in about 20 h. Raising the temperature can slightly accelerate the initial adsorption rate and slightly increase the final equilibrium adsorption, which implies that the adsorption process may be a slight heat adsorption process, which is consistent with the physical adsorption process.

To evaluate the practical applicability of CINPs@MXene/PU/PDMS sponge, we examined its adsorption performance on 100 nm PS NPs in a simulated complex water environment. Figure 7D shows that compared with the removal efficiency in deionized water, the inhibitory effect of 50% (*v*/*v*) ethanol was the most significant, and the adsorption capacity was reduced to about 70.04 mg/g, which is mainly due to its significant change in the dielectric constant of the solvent, thus greatly weakening the hydrophobic interaction. SiO_2_, Pb^2+^ and methylene blue also show moderate inhibition (Q_e_ was between 122 and 137 mg/g), which may be related to competitive adsorption and physical blockage.

To determine the control steps of the adsorption rate, we have fitted the kinetic data. The results clearly show that the PFO model (R^2^ = 0.976) was better than the PSO model (R^2^ = 0.959), and its calculated equilibrium adsorption (160.1 mg/g) was highly consistent with the experimental value (152.5 mg/g). This strongly proves that the adsorption rate is mainly controlled by the diffusion step, which is a typical physical adsorption characteristic.

The fitting results of the W–B model and the Elovich model further revealed the mass transfer mechanism. W–B fitting shows obvious multi-stage linear characteristics and was not the origin, confirming that adsorption was dominated by the initial external liquid membrane diffusion and the subsequent rate-limiting step-internal pore diffusion. At the same time, the good fit of the Elovich model (R^2^ = 0.925) shows that the physical adsorption of this diffusion control occurs on a heterogeneous surface with uneven energy, which was completely consistent with the complex structure of our composite sponge.

Adsorption isotherm analysis shows that the Freundlich model (R^2^ = 0.969) can describe the adsorption behavior more accurately than the Langmuir model (R^2^ = 0.967). The core hypothesis of the Freundlich model (uneven surface) was highly consistent with the physical properties of our material. Its heterogeneity parameter 1/n was 0.633, which confirms the high unevenness of the adsorption surface. Therefore, it can be determined that the adsorption process was a multimolecular physical adsorption process that occurs on heterogeneous surfaces.

To further elucidate the specific contribution of each component to the adsorption mechanism, a comparative experiment was conducted using the intermediate CINPs@MXene/PU sponge (without PDMS coating). As shown in Figure S6, the equilibrium adsorption capacity of the CINPs@MXene/PU sponge for 100 nm PS NPs was approximately 76 mg/g after 20 h. In stark contrast, the final CINPs@MXene/PU/PDMS sponge achieved a significantly higher capacity of 152.5 mg/g under identical conditions. This nearly two-fold enhancement strongly confirms that while the rough CINPs@MXene/PU skeleton provides physical entrapment sites, the low-surface-energy PDMS coating plays the dominant role in capturing hydrophobic nanoplastics via strong hydrophobic interactions.

Figure 7H shows that CINPs@MXene/PU/PDMS sponges show efficient and stable microplastic removal ability and good reusability in a variety of actual water bodies and complex media. Although the high-salinity environment (such as water in the South China Sea) will slightly reduce the removal efficiency due to the ion intensity effect, in complex media containing a large amount of oil (such as takeout soup), the sponge can significantly improve the removal efficiency of microplastics by adsorbing oil and water at the same time, and using the wrapping effect of oil on microplastics. These results fully prove the application potential of this material in different practical environments.

## 4. Discussions

To simultaneously address the complex challenge of oil and microplastic pollution in water, this study presents a novel CINPs@MXene/PU/PDMS sponge developed through a well-devised synergistic strategy. Our results demonstrate that this multifunctional platform is not only highly efficient but also structurally robust, chemically stable, and safe for practical applications.

The exceptional performance of this composite sponge originates from its rational, multi-layered design. We utilized waste polyurethane (PU) sponges as the foundational scaffold. The critical step in building its core functionality was the deposition of the CINPs@MXene composite, which not only created the micro/nanoscale roughness essential for hydrophobicity but also laid the groundwork for its subsequent multifunctional properties. Finally, a sealing coat of low-surface-energy PDMS was applied to drastically lower the surface energy. It is this elaborate structural design that endows the sponge with its excellent oil–water selectivity and high absorption capacity for various oils and organic compounds.

A key innovation of this work is the integration of a solar-driven photothermal effect to tackle the challenging issue of high-viscosity crude oil. The potent combination of inexpensive, melanin-like CINPs and MXene nanosheets creates a powerful “photothermal engine,” capable of rapidly heating the sponge’s surface to 84.1 °C within seconds under simulated sunlight. This localized and rapid heating is not merely a physical phenomenon but a highly effective functional tool—it dramatically reduces the viscosity of viscous crude oil, thereby greatly accelerating its permeation and adsorption. This is compellingly demonstrated by the experimental data, where the saturated adsorption capacity for crude oil leaped from about 9 g/g in the absence of light to nearly 25 g/g with light assistance. This strategy offers a sustainable, low-energy solution for viscous oil spills, representing a significant breakthrough compared to conventional adsorbents.

Beyond its exceptional oil removal capabilities, the sponge also exhibits a strong, broad-spectrum adsorption capacity for common microplastics and nanoplastics. To contextualize the performance of our material, it is worth comparing it with recently reported dual-functional adsorbents. For instance, Quan et al. [48] developed a biodegradable loofah sponge that achieved high oil absorption and microplastic capture; however, it lacked the photothermal capability necessary for the rapid cleanup of high-viscosity crude oil. Similarly, while Xue et al. [49] reported a photothermal TiO_2_/PDMS sponge effective for oil and microplastics, their work primarily focused on larger-sized microplastics. In contrast, our CINPs@MXene/PU/PDMS sponge distinguishes itself by integrating biomass-derived photothermal components to efficiently handle high-viscosity oil while simultaneously demonstrating the effective capture of much smaller 100 nm nanoplastics, addressing a more challenging class of pollutants. The adsorption is primarily driven by hydrophobic interactions, characteristic of a physical adsorption process. An intriguing phenomenon was observed in complex, oily aqueous media, such as simulated “takeout soup,” where the removal efficiency for microplastics was significantly enhanced. We attribute this to a synergistic “wrapping” effect: the absorbed oil phase acts to further capture and sequester microplastic particles. This finding powerfully demonstrates the material’s immense potential for treating real-world, complex wastewater.

Ultimately, the value of any environmental remediation material is determined by its practical reliability, and in this regard, the modified sponge also excels. The incorporation of MXene significantly enhances the material’s mechanical strength, enabling it to withstand at least 500 compression cycles. Its superhydrophobic surface remains remarkably stable against harsh acidic and alkaline conditions, in various natural water bodies, and after repeated physical abrasion. Crucially, our design successfully addresses the long-standing flammability risk associated with PU-based materials. Both TGA and Limiting Oxygen Index (LOI) tests (showing an increase from 17.5% to about 26%) confirm that the composite sponge possesses far superior thermal stability and flame retardancy than pristine PU. Its self-extinguishing behavior upon flame contact is a critical performance enhancement.

In summary, this work presents not only a high-performance multifunctional adsorbent but also puts forward a scalable design strategy. By synergistically combining biomass-derived nanomaterials with 2D materials, it is possible to develop more efficient and reliable solutions to address the increasingly complex and severe environmental challenges we face.

## 5. Conclusions

In a word, the CINPs@MXene/PU/PDMS sponge has successfully integrated ultra-hydrophobic, high-efficiency oil absorption and oil–water separation, photothermal auxiliary oil recovery, broad-spectrum microplastic adsorption, and enhanced flame retardancy. At 1.5 times the intensity of sunlight, its surface temperature can rise to 84.1 °C, and the photothermal effect remained stable after five cycles. The sponge maintained stable ultra-hydrophobic and excellent reusability, and can still maintain more than 95% of its initial adsorption capacity after 20 cycles. Its oil–water separation efficiency was as high as 97%. For high-viscosity crude oil, the saturation absorption was as high as 25 g/g at 1.5 times the sunlight intensity, and at 1.0 times the sunlight intensity, crude oil can be quickly absorbed in just in 4 min, achieving a removal rate of 91.4%. More importantly, it has stable mechanical strength and excellent flame-retardant properties, and still maintains hydrophobic stability in a variety of harsh environments. It shows broad-spectrum microplastic adsorption ability, has a super adsorption ability on nanoplastics, and its main adsorption mechanism is physical adsorption. In the complex practical environment, it still maintained a stable adsorption ability for nanoplastics and had not significantly weakened by multiple cycles. These comprehensive properties make the sponge a promising candidate material for environmental restoration, emergency treatment of oil spillage, and water purification.

## Figures and Tables

**Table 1:** The loading amount of CINPs@MXene and its naming.

Weight Percentage of CINPs@MXene in DMF	Naming
0.2	0.2CINPs@MXene/PU/PDMS
0.4	0.4CINPs@MXene/PU/PDMS
0.6	0.6CINPs@MXene/PU/PDMS
0.8	0.8CINPs@MXene/PU/PDMS

## Data Availability

The original contributions presented in the study are included in the article. Further inquiries can be directed to the corresponding author.

## Supplementary Materials

The following supporting information can be downloaded at https://www.mdpi.com/article/10.3390/polym18030324/s1. Figure S1. The particle size distribution obtained by TEM measurement in CINPs. Figure S2. Map Sum Spectrum of CINPs@MXene/PU/PDMS sponge. Figure S3. WCA of PU/PDMS Sponge. Figure S4. Surface morphology of CINPs@MXene/PU/PDMS sponge after microplastic adsorption (PET, 300 nm). Figure S5. Calibration curves of the concentrations of PS NPs (100 nm) versus corresponding fluorescence intensities (Wavenumber (Ex/Em): 532 nm/585 nm). Figure S6. The adsorption capacity of CINPs@MXene/PU sponge for PS NPs. Figure S7. DTG of PU sponge and CINPs@MXene/PU/PDMS sponge. Figure S8. Oxidant situation of CINPs@MXene and MXene. Table S1. The elemental content of CINPs@MXene/PU/PDMS sponge. Video S1. Water droplets rolling down. Video S2. Modified sponge absorbing heavy oil. Video S3. Modified sponge absorbing light oil.
