# Supplementary material for: MXene/Cuttlefish-Ink Nanoparticles Incorporated Dual-Purification Sponge for Solar-Driven Oily Wastewater and Microplastic Remediation
Source: Polymers (Basel). 2026 Jan 26;18(3):324. doi: 10.3390/polym18030324 (PMC12899509; doi:10.3390/polym18030324)
Supplement: Supplementary file 1 [file polymers-18-00324-s001.zip › Supplementary material.pdf]

Support Information

MXene/Cuttlefish-Ink Nanoparticles Incorporated Dual-Purification Sponge for Solar-Driven Oily Wastewater and Microplastic Remediation

Huixuan Sun [1,2], Qirui Gong [3], Lihong Fan [1,2,3,*], Shilin Tian [3], Shiyuan Yao [1,3], Guangxu Wang [3], Sasha You [3] and Wei Zhang [3]

[1] Sanya Science and Education Innovation Park of Wuhan University of Technology, Sanya 572024, China
[2] School of Resources and Environmental Engineering, Wuhan University of Technology, Wuhan 430070, China
[3] School of Chemistry, Chemical Engineering and Life Sciences, Wuhan University of Technology, Wuhan 430070, China
* Correspondence: lhfan@whut.edu.cn

1. Experimental Section

1.1 Oxidation situation of CINPs@MXene and MXene

To evaluate the antioxidation stability, CINPs@MXene composites (with a mass ratio of CINPs to MXene = 3:1) and pristine MXene were placed in an oven at 45 °C for 15 days. After the aging process, the samples were collected and their morphological structures were examined using transmission electron microscopy (TEM).

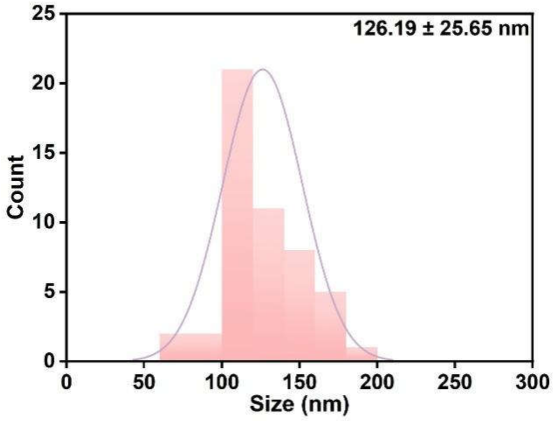

Figure S1. The particle size distribution obtained by TEM measurement in CINPs

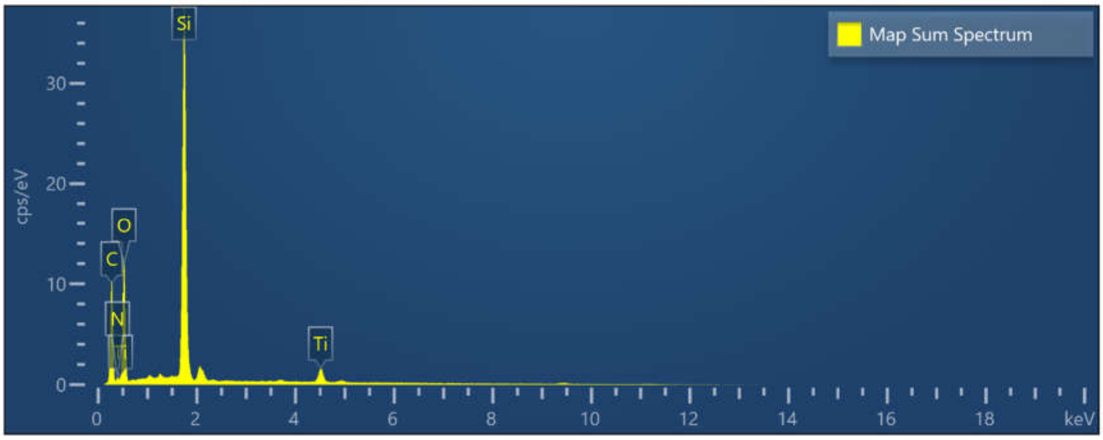

Figure S2. Map Sum Spectrum of CINPs@MXene/PU/PDMS sponge

Table S1. The elemental content of CINPs@MXene/PU/PDMS sponge

Element	Weight (%)	Atomic(%)
C	53.83	63.84
N	2.44	2.48
O	30.98	27.56
Si	11.16	5.65
Ti	1.58	0.47
Total	100%	100%

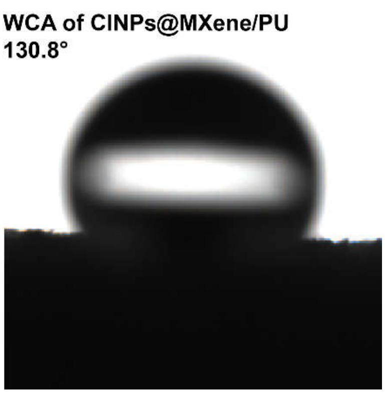

Figure S3. WCA of PU/PDMS sponge

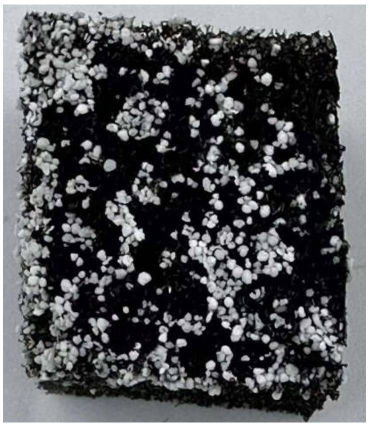

Figure S4. Surface morphology of CINPs@MXene/PU/PDMS sponge after microplastic adsorption (PET, 300 nm)

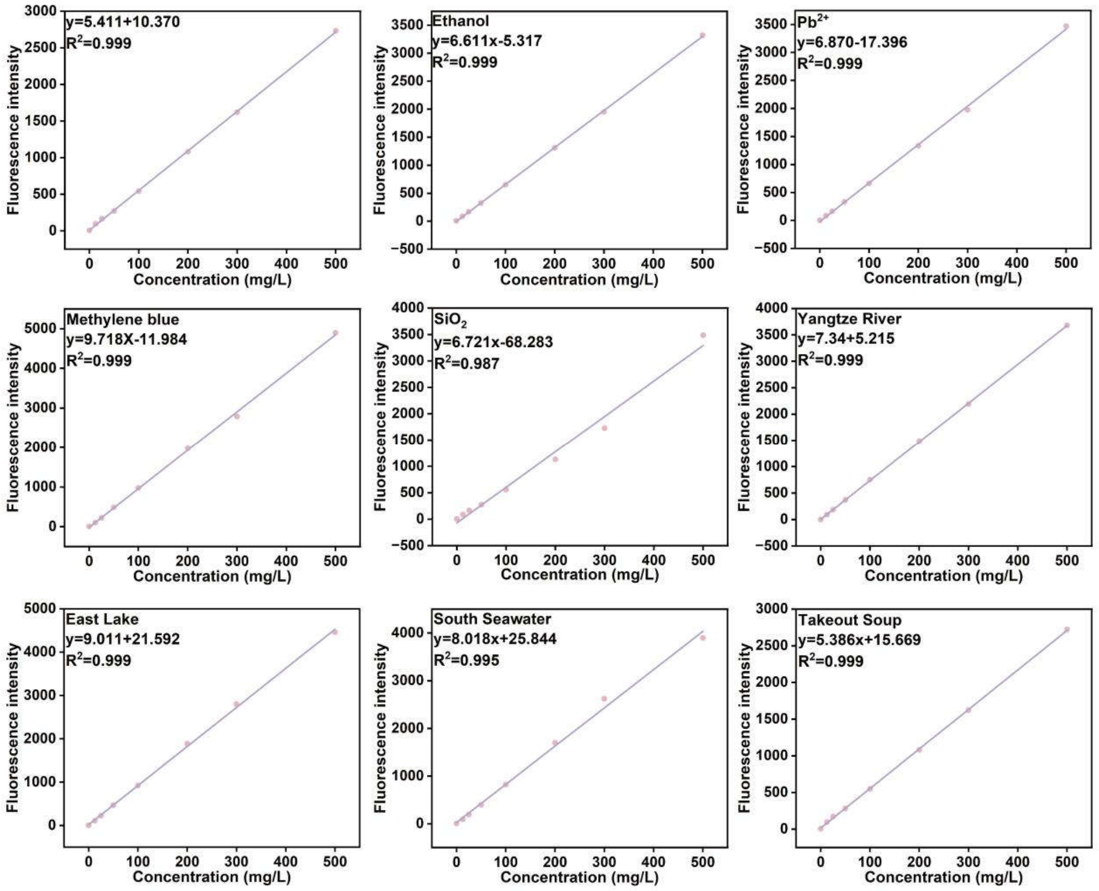

Figure S5. Calibration curves of the concentrations of PS NPs (100 nm) versus corresponding fluorescence intensities (Wavenumber (Ex/Em): 532 nm/585 nm).

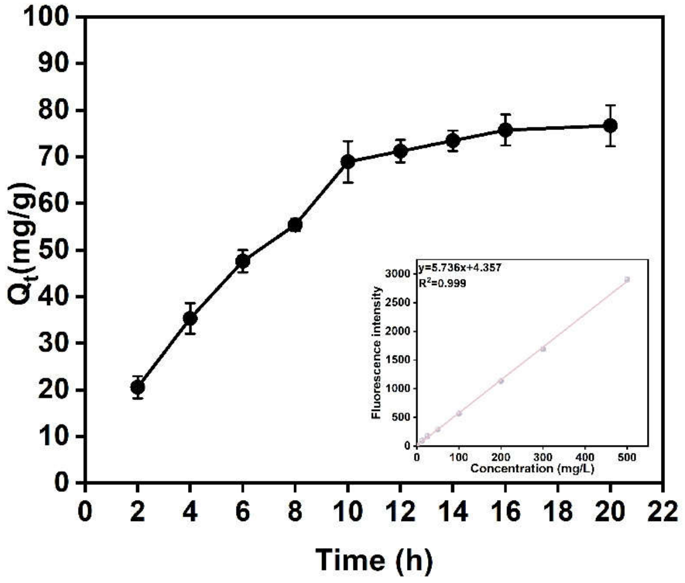

Figure S6. The adsorption capacity of CINPs@MXene/PU sponge for PS NPs

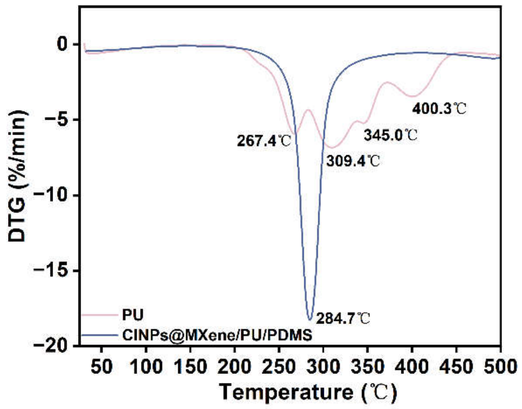

Figure S7. DTG of PU sponge and CINPs@MXene/PU/PDMS sponge

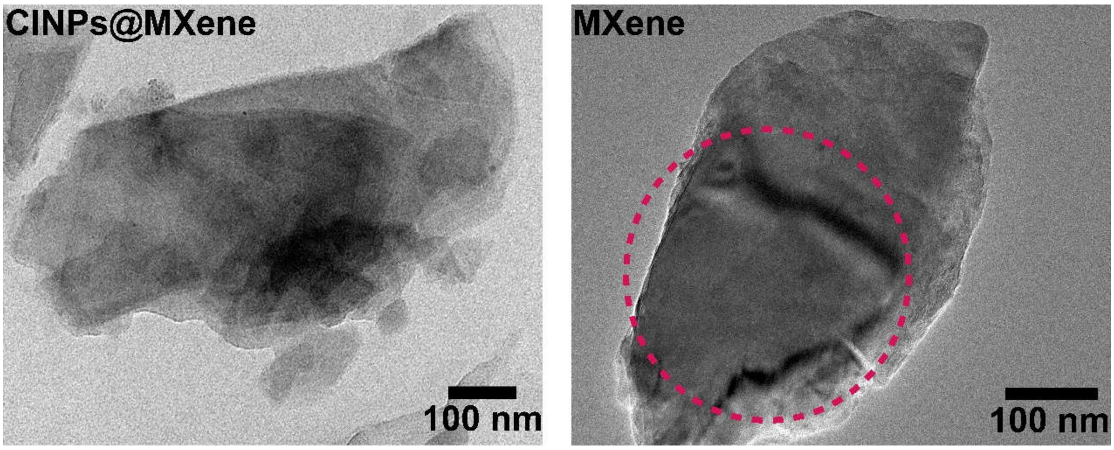

Figure S8. Oxidant situation of CINPs@MXene and MXene
